# Unlocking a dark past

**DOI:** 10.7554/eLife.41002

**Published:** 2018-09-25

**Authors:** Fernando Rodríguez-Pérez, Michael Rape

**Affiliations:** 1 Howard Hughes Medical InstituteUniversity of CaliforniaBerkeleyUnited States; 2 Department of Molecular and Cell BiologyUniversity of CaliforniaBerkeleyUnited States

**Keywords:** Chemical Biology, Teratogenicity, Ubiquitin, Transcription Factors, Thalidomide, Human

## Abstract

A transcription factor called SALL4 could be the missing link between thalidomide and the limb defects caused by the drug.

**Related research article** Donovan KA, An J, Nowak RP, Yuan JC, Fink EC, Berry BC, Ebert BL, Fischer ES. 2018. Thalidomide promotes degradation of SALL4, a transcription factor implicated in Duane Radial Ray Syndrome. *eLife* **7**:e38430. doi: 10.7554/eLife.38430

On October 2, 1957 the German pharmaceutical company Grünenthal introduced Contergan, a new over-the-counter drug to treat insomnia. Grünenthal researchers had originally obtained its active compound, thalidomide, by heating a commercially available chemical (phthaloyl isoglutamine) in an attempt to develop a new antibiotic (Brynner and Stephens, 2001). However, after failing to observe any antibiotic activity, Grünenthal searched for other possible applications and found that thalidomide induced sleep in about 60% of treated patients. Although this sleep-inducing effect was not observed in mice and rats, the rodents tolerated very high levels of the drug, prompting Grünenthal to proclaim that the new sedative was completely safe and could even be taken by pregnant women for morning sickness.

This turned out to be a dramatic mistake: repeated use of thalidomide induced peripheral neuropathy, and if taken during the first trimester of pregnancy, even a single dose triggered birth defects referred to as phocomelia, a reduction or absence of limbs. When Contergan was finally removed from the shelves in 1961, it may have caused birth defects in nearly 10,000 individuals, approximately 5,000 of whom survived childhood. Yet, exactly how thalidomide causes limb deformations in humans, while also sparing rodents, has remained a mystery (Lenz, 1988).

Despite its dark past, thalidomide and its derivatives, lenalidomide and pomalidomide, found their way back into the clinic. Commonly referred to as immunomodulatory drugs, these compounds are used as first-line treatment for multiple myeloma, del(5q)-MDS and leprosy. Similar to the role of thalidomide in causing birth defects, the biological mechanism underlying its therapeutic benefits had been unknown until 2010, when the primary binding target of thalidomide was identified as a protein called cereblon (CRBN; Ito et al., 2010). This protein forms a complex called CUL4^CRBN^ (Figure 1), which is known to be an E3 ubiquitin ligase.

In multiple myeloma, rather than inhibiting this ligase, thalidomide recruits two zinc-finger transcription factors to the complex, which results in these transcription factors being ubiquitylated and subsequently degraded by proteasomes (Lu et al., 2014). A similar mechanism operates in del(5q)-MDS, where the drugs trigger the degradation of a kinase called CK1α (Krönke et al., 2015; Petzold et al., 2016). However, neither of these neo-substrates shed light on the teratogenic effects of thalidomide, and the question of how the drug interfered with limb development remained unanswered. Now, in eLife, Eric Fischer and colleagues at the Dana-Farber Cancer Institute, Harvard Medical School and Brigham and Women's Hospital – including Katherine Donovan as first author – report results that shed light onto this decades-old question (Donovan et al., 2018).

**Figure 1.**
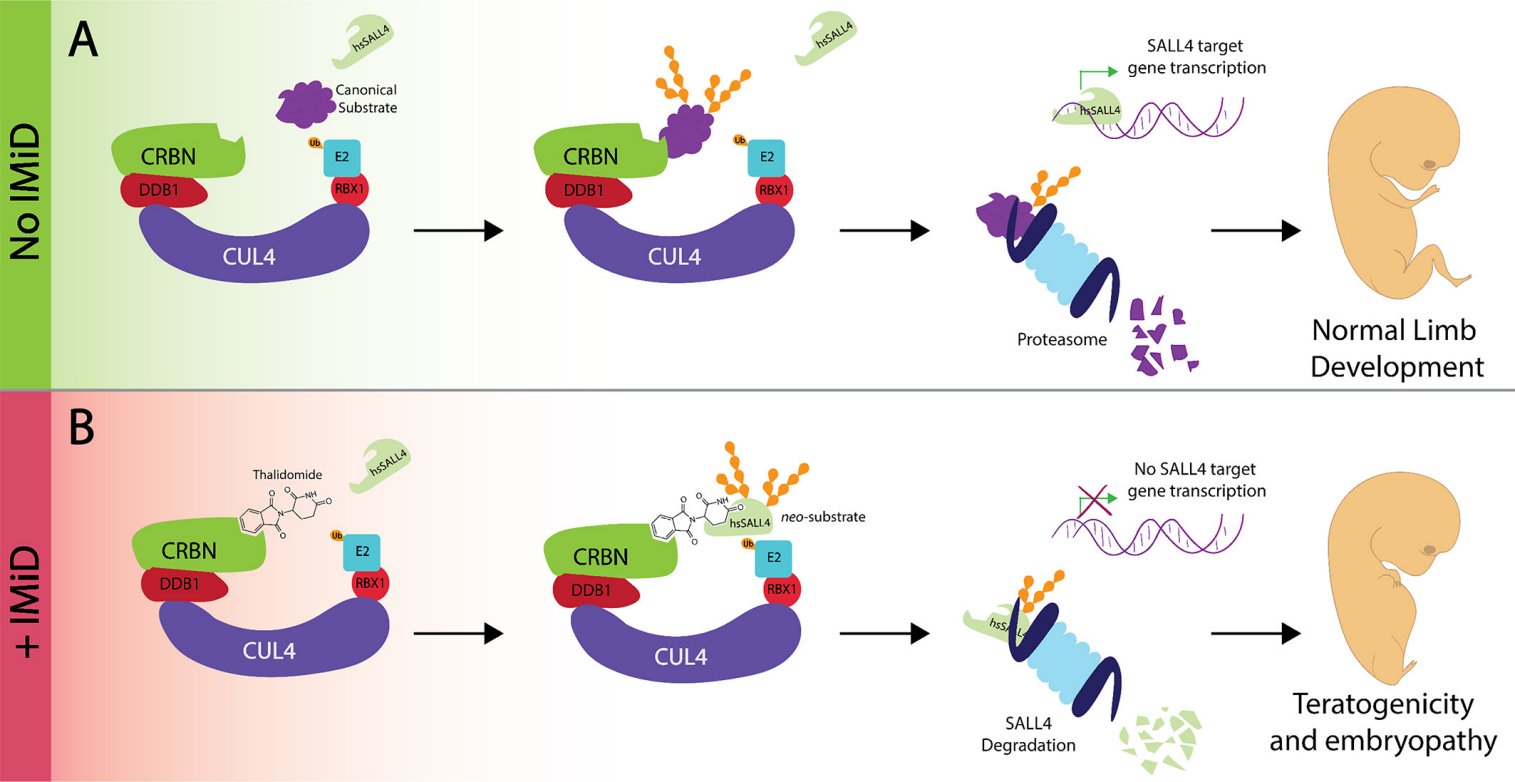
Thalidomide, the transcription factor SALL4 and limb defects. (**A**) In the absence of an immunomodulatory drug like thalidomide the CUL4^CRBN^ complex (multiple colors) 'tags' a substrate (purple) for degradation by the proteasome (pale blue). SALL4 remains intact during this process and is therefore able to control the transcriptional activity of target genes to control robust embryonic limb development. (**B**) The presence of thalidomide results in SALL4 being tagged for degradation. The subsequent absence of SALL4 prevents the transcription of its target genes and thus likely interferes with limb development, as observed in thalidomide syndrome. iMiD: immunomodulatory drug.

Human embryonic stem cells are powerful models in which to study the regulatory circuits of human development. Donovan et al. used quantitative mass spectrometry in such stem cells to identify proteins that were degraded upon treatment with thalidomide, lenalidomide or pomalidomide. While they found several proteins that were degraded by one or two of these drugs, they found only one protein that was degraded by all three: this was a transcription factor called SALL4. SALL4 behaved like other canonical substrates of thalidomide and the CUL4^CRBN^ complex, with the first two zinc fingers being essential for the complex to recognize SALL4 when the drug is present. Moreover, the mutation of a glycine residue in one of the zinc-finger transcription factors was sufficient to disrupt this drug-induced association (Petzold et al., 2016).

Multiple observations suggest that identifying SALL4 as a substrate for the CUL4^CRBN^ complex is an important step on the road to understanding how thalidomide triggers phocomelia. First, SALL4 drives limb development in metazoan organisms (Koshiba-Takeuchi et al., 2006); Figure 1A). Moreover, mutations in the gene for SALL4 cause Duane Radial Ray Syndrome and Holt-Oram Syndrome, two diseases in which limbs are deformed: indeed, carriers of these mutations have been misdiagnosed as victims of thalidomide (Borozdin et al., 2004; Kohlhase et al., 2002; Kohlhase et al., 2003). While heterozygous mutations cause the two syndromes, homozygous mutations are never observed in patients, and deleting both alleles is lethal in mice. Taken together, these observations imply that partial degradation of SALL4, as accomplished by thalidomide, could be consistent with life, yet induce phocomelia (Figure 1B).

It was known that CRBN variants that are sensitive to thalidomide, such as those carried by monkeys and humans, contain the amino acid valine at position 388. This is required for substrates to bind to the CUL4^CRBN^ complex in the presence of the drug, and Donovan et al. show that, in human CRBN, valine is necessary for SALL4 to be tagged for degradation by CUL4^CRBN^. By contrast, most species resistant to thalidomide contain the amino acid isoleucine at this position, which prevents the recruitment of new substrates to CRBN in presence of the drug or its derivatives.

However, mice expressing a humanized CRBN with valine in position 388 do not show limb defects upon thalidomide treatment. Accordingly, changing isoleucine to valine in position 388 in CRBN was not sufficient to induce the degradation of SALL4. There are, therefore, other differences in the sequences of SALL4 variants in rodents which protect these animals from the toxic effects of thalidomide. Indeed, when Donavan et al. combined human CRBN with a ‘humanized’ variant of mouse SALL4 that contained five mutations in the critical second zinc finger, they observed a degradation of mouse SALL4 under the effect of the drug. These results strongly point towards the degradation of SALL4 having a role in thalidomide being toxic for embryos. They also highlight the challenges involved in using mouse models to evaluate drug candidates for clinical use. As such, they exemplify the importance of understanding drug-target interactions at a molecular level before introducing new therapeutics into the market.

Experiments are now required that will combine the expression of humanized SALL4 and CRBN in a thalidomide-resistant species, such as mice. If thalidomide creates defects in limb development in this model, like it did in way too many children, it would bring proof that the degradation of SALL4 leads to phocomelia when the drug is present. While the destruction of SALL4 likely contributes to the emergence of phocomelia, thalidomide and its related compounds also induce the degradation of many other transcription factors, which raises the possibility that additional targets might also play a role in the toxic effects of thalidomide on embryos. Humanized SALL4-animals might provide a starting platform to discover these missing targets, particularly those that are related to other symptoms such as damage in peripheral nerves.

Importantly, thalidomide has recently gained much attention for its use as a proteolysis targeting chimera (PROTAC): a PROTAC is a small molecule that targets disease-causing proteins to a complex (such as CUL4^CRBN^) for ubiquitylation and degradation (Churcher, 2018; Sakamoto et al., 2001). Yet, using PROTACs based on thalidomide runs into the danger of interfering with limb development. Companies are therefore hoping to find and develop other molecules that recruit proteins to CUL4^CRBN^ without having toxic effects on human development. The discovery of SALL4 as a target for thalidomide sheds new light on a man-made pharmaceutical disaster, and may make it possible to use thalidomide more safely.
